# “Disruptive Behavior” or “Expected Benefit” Are Rationales of Seclusion Without Prior Aggression

**DOI:** 10.3389/fpsyt.2022.871525

**Published:** 2022-04-15

**Authors:** Fleur J. Vruwink, Joanneke E. L. VanDerNagel, Eric O. Noorthoorn, Henk L. I. Nijman, Cornelis L. Mulder

**Affiliations:** ^1^Mediant Geestelijke Gezondheidszorg (GGZ), Enschede, Netherlands; ^2^Tactus, Deventer, Netherlands; ^3^Department of Human Media Interactions, University of Twente, Enschede, Netherlands; ^4^Aveleijn, Borne, Netherlands; ^5^Nijmegen Institute for Scientist-Practitioners in Addiction, Radboud Universiteit Nijmegen, Nijmegen, Netherlands; ^6^GGNet Geestelijke Gezondheidszorg, Warnsveld, Netherlands; ^7^Clinical Psychology, Department of Social Sciences, Behavioural Science Institute (BSI), Radboud University, Nijmegen, Netherlands; ^8^Department of Psychiatry, Epidemiologic and Social Psychiatry Research Institute (ESPRI), Erasmus MC, Rotterdam, Netherlands

**Keywords:** psychiatry, seclusion, aggression, involuntary treatment, seclusion reduction, involuntary hospitalization, reasons for seclusion

## Abstract

**Objective:**

In the Netherlands, seclusion of patients with a psychiatric disorder is a last-resort measure to be used only in the event of (imminent) severe danger or harm. Although aggressive behavior is often involved, seclusions not preceded by aggression also seem to occur. We sought insight into the non-aggressive reasons underlying seclusion and investigated the factors associated with it.

**Method:**

We included all patients admitted to a Dutch psychiatric hospital in 2008 and 2009. Seclusions had been registered on Argus-forms, and aggression incidents had been registered on the Staff Observation Aggression Scale-Revised (SOAS-R), inspectorate forms and/or patient files. Determinants of seclusion with vs. without prior aggression were analyzed using logistic regression. Reasons for seclusion without prior aggression were evaluated qualitatively and grouped into main themes.

**Results:**

Of 1,106 admitted patients, 184 (17%) were secluded at some time during admission. Twenty-one (11.4%) were excluded because information on their seclusion was lacking. In 23 cases (14%), neither SOAS-R, inspectorate forms nor individual patient files indicated any aggression. Univariable and multivariable regression both showed seclusion without preceding aggression to be negatively associated with daytime and the first day of hospitalization. In other words, seclusion related to aggression occurred more on the first day, and during daytime, while seclusion for non-aggressive reasons occurred relatively more after the first day, and during nighttime. Our qualitative findings showed two main themes of non-aggressive reasons for seclusion: “disruptive behavior” and “beneficial to patient.”

**Conclusion:**

Awareness of the different reasons for seclusion may improve interventions on reducing its use. Thorough examination of different sources showed that few seclusions had not been preceded by aggression. The use of seclusion would be considerably reduced through interventions that prevent aggression or handle aggression incidents in other ways than seclusion. However, attention should also be paid to the remaining reasons for seclusion, such as handling disruptive behavior and focusing on the beneficial effects of reduced stimuli. Future research on interventions to reduce the use of seclusion should not only aim to reduce seclusion but should also establish whether seclusions preceded by aggression decrease different from seclusions that are not preceded by aggression.

## Introduction

Seclusion, defined as solitary confinement of patients, is viewed as a coercive strategy that can have severe negative side-effects for both the psychiatric patients and staff involved in it (1–6), but some believe patients can also benefit from it (7–9). Despite policies to reduce its number and duration, it continues to be used frequently in mental health services around the world (10–14).

Under the applicable mental health law, seclusion and involuntary medication is permitted in the Netherlands only as a last resort (15, 16). Involuntary treatment or placement may be justified in connection with a mental disorder of a serious nature, if from the absence of treatment or placement serious harm is likely to result to the person’s health or to a third party (17). Aggressive behavior or the threat of aggression are commonly accepted indications for using seclusion and restraint (2, 6, 18, 19).

Several studies have shown that approximately half the number of seclusions (range 12–100%) was indeed preceded by (imminent) aggressive incidents (13, 19–28). This also implies that roughly half (i.e., 0–88%) was NOT preceded by (imminent) aggression.

Agitation without clear aggression has been found to be a common reason for seclusion or restraint (2, 19, 20, 23, 27, 29, 30). Two other commonly reported non-violent reasons were disruptive or disturbed patient behavior (20, 30, 31), and risk of absconding (31, 32). Less commonly reported reasons included uncooperativeness (33), psychotic or delusional episodes, intoxicated behavior (20), and reduction of stimuli (2, 20).

The widely ranging percentages of seclusion preceded by aggression highlight large differences between studies, hospitals and wards [e.g., (20, 21, 28)]. In centers with the highest rates of seclusion and restraint, Betemps et al. (20) found that these measures were motivated more by agitation than they were in centers with lower rates. However, the inverse relationship was found for “disruptive or disturbed patient behavior”: in centers with lower rates seclusion was motivated more by this behavior than they were in centers with higher rates (20).

Authors, including Brown et al. (29) and Kaltiala Heino et al. (19) have questioned the necessity of seclusion or other coercive measures for non-violent reasons, because the most common reasons found by these authors were patients’ agitation and/or disorientation unaccompanied by evidence of actual or threatening violence to persons or even to property (19, 29). On the other hand, not all aggressive patients were secluded, although the violence was as severe as that in the patients who were secluded (29).

Due to the negative consequences for the psychiatric patients and staff involved, reductions in the use of seclusion are being attempted at an international level (34). However, these attempts pay little attention to the distinction between seclusion in response to aggression and seclusion without prior aggression. Failure to examine seclusions without preceding aggression may obstruct its reduction in practice. Happell and Harrow (35) pointed out, if seclusion is to be reduced, it is crucial to understand the patterns of its use, including recognition of the characteristics of secluded patients, and enhanced knowledge about the types of patient who are more likely to experience seclusion. Such understanding provides vital information that can be used to tailor and implement seclusion-reduction interventions (35).

To be able to develop such interventions, greater knowledge is needed of the differences between seclusion with and without prior aggression and the details of the reasons for seclusion. To our knowledge, no studies have been published on the patient-related factors that distinguish between these types of seclusion. We therefore investigated the differences between patients whose seclusion had and had not been preceded by aggression, and also examined the reasons for non-aggressive seclusion stated in the patient files. We specifically wished to establish the following:

1. How often patients had been secluded for reasons other than aggression.
2. The patient-related factors associated with seclusion with vs. without prior aggression, and
3. The reasons for the use of seclusion without preceding aggression.

## Materials and Methods

### Design

We used a mixed method (36) combining both qualitative and quantitative data to categorize the cases into APS and NAPS. We continued the analyses first with a quantitative part, followed by a qualitative part. The quantitative part used logistic regression modeling to analyze data on seclusion and aggression. The qualitative part used text fragments from patient files to gain insight into the reasons patients had been secluded without preceding aggression.

Under Dutch law this research design is exempt from medical ethical review (37), a fact that was affirmed by the Southern Chamber of the Dutch Ethics Review Board.

### Setting and Inclusion

We collected the data of patients admitted to a 265-bed Dutch mental health trust located in a predominantly rural catchment area with 400,000 inhabitants in the eastern Netherlands. A total of 16 wards were located at 4 individual sites. Ten of these were open and six were closed wards; twelve wards were for adults and four for elderly patients (60+ years). All closed and three open wards had one or more seclusion rooms. We included all patients who had been secluded between 1 January 2008 and 31 December 2009. To avoid disproportionate contributions by patients who had been secluded more than once, we used data only on each patient’s first seclusion in the study period.

### Measurements

#### Demographic, Diagnostic, Mental Health History, and Contextual Data

From the hospital’s database we took not only patients’ demographic and diagnostic data, which included age, gender, country of birth [Western or non-Western (38)], marital status, and mental health diagnoses; but also admission data including date of admission, duration of hospitalization, previous admissions, involuntary legal status during hospitalization, and type of ward (open or closed ward, and acute or longstay ward). As involuntary seclusion in the Netherlands needs to be accompanied by an involuntary admission we choose to analyze the juridical status 1 day before the seclusion.

#### Seclusion

Seclusion was defined as solitary confinement in a seclusion room without the option of leaving it. Dutch seclusion rooms have to fulfill government criteria (39), such as minimum size, access to basic sanitary facilities, provisions for communication between staff and secluded patients; and smoothly plastered walls and smoothly finished floors. In the Netherlands seclusion can occur with consent of the patient, but at least half is used as a coercive measure (40).

To register all coercion-episodes, including seclusion, nurses used Argus forms, which were mandatory. Nurses reported each coercive measure for each day separately, recording the times of onset and termination for all patients, regardless of the legal status (voluntary or coercive admission), and whether or not a patient had objected to the use of the coercive measure (41). This study covered all seclusions, both with and without consent.

#### Aggression

According to the definition used in the Staff Observation Aggression Scale–revised (SOAS-R) (42), aggression was defined as any verbal, non-verbal, or physical behavior that was threatening to self, others or property; or as physical behavior that actually did harm to self, others, or property. By itself, agitation was not considered to be a form of aggression. The outcome variable was either aggression preceding seclusion (APS) or no aggression preceding seclusion (NAPS).

To ensure that seclusions preceded by aggressive behavior (APS) were not falsely classified as seclusion not preceded by aggression (NAPS), aggression was measured in three ways:

1. SOAS-R: Data on aggression incidents were gathered using the SOAS-R (42), which had been part of the incident reporting system at this mental health trust since 2003. After each incident of aggression, a staff member who witnessed it—usually a nurse—completed the SOAS-R form stating the location, date, and nature of the incident. The SOAS-R comprises five columns pertaining to specific aspects of aggressive behavior: (1) the provocation; (2) the means used by the aggressor; (3) the target of aggression; (4) the consequence or consequences for victim or victims; and (5) the measure or measures taken to stop aggression. We viewed the following as the reason for seclusion: the fact that the SOAS-R form had been filled out, identifying the patient in question as the aggressor on the date of his his/her seclusion. SOAS-R forms from before the date of seclusion were considered to be “aggression incidents in the patient’s history.”
2. Inspectorate forms: Under Dutch law the start of all forced treatments and restrictive measures must be reported to the Dutch Health Care Inspectorate. Forms designed for this purpose should inform the inspectorate which coercive measures would be used over a period of time with the patient in question. Unlike the Argus forms, which register the precise time a measure is applied, these notification forms specify the reason or reasons for using coercive measures. Copies of these forms are kept in the archives of the hospital concerned. Working to the definition of aggression given in the passage above, two authors with experience in psychiatric care (FV and EN) independently checked these forms for (imminent) aggression. In the event of disagreement between them, consensus was achieved by discussion.
3. Patient files: Finally, for references to aggression, we also checked the patient files of all included patients who, on the day of seclusion, had no entry on the SOAS-R form; or no mention of aggression on the inspectorate form. Patient files contain the daily notes of nurses, doctors, and other staff. If these notes mentioned or described aggression in relation to the subsequent seclusion, this case was considered to be APS. The same two researchers (FV and EN) scored the notes independently as APS or NAPS. Cases that had been appraised differently were discussed before finally being classified.

In brief, when seclusion was preceded by what one or more of these sources had referred to as patient aggression, we defined it as having been “preceded by aggression” (APS). All other seclusions were considered not to have been preceded by aggression (NAPS).

#### Non-violent Reasons for Seclusion

The files of NAPS patients were then studied in detail by two authors (FV and JV), who, seeking possible reasons for seclusion, looked for information on individual patients behavior up to 24 h before seclusion started. Relevant text fragments illustrating reasons for seclusion were separately coded and extracted from the files. If applicable, several reasons could be attributed to one seclusion episode.

### Data Analyses

#### Statistical Analyses to Compare Determinants of No Aggression Preceding Seclusion vs. Aggression Preceding Seclusion

Using IBM SPSS Statistics 26, we performed univariable logistic regression to investigate which factors, grouped into demographic, diagnostic, historical and contextual factors, discriminated between NAPS and APS. Secondly, we used multivariable regression analyses to correct the univariable factors for each other. As recommended when building models for regression (43), we included the variables that were associated with NAPS with a *p*-value <0.20 in the univariable analyses. The alpha level was set at 5%.

#### Analyses of Patient Files

From the daily notes in the EPF we selected text fragments relevant to identify a reason for seclusion. These text fragments were analyzed, using MaxQDA software (VERBI Software GmbH, Berlin, Germany) for qualitative data analysis (44). By consensus, the fragments were grouped, and if necessary regrouped, and subsequently labeled into main themes and subthemes by two clinicians (FV and JV) who thus developed a framework of reasons for NAPS. Below, these themes are illustrated by citations from the notes. In this analysis we included all cases. However, due to the limited number of cases saturation was not obtained.

## Results

### Number of Seclusions Preceded by Aggression

In our sample of 1,106 patients 184 (16.6%) unique patients had been secluded.

**FIGURE 1:**
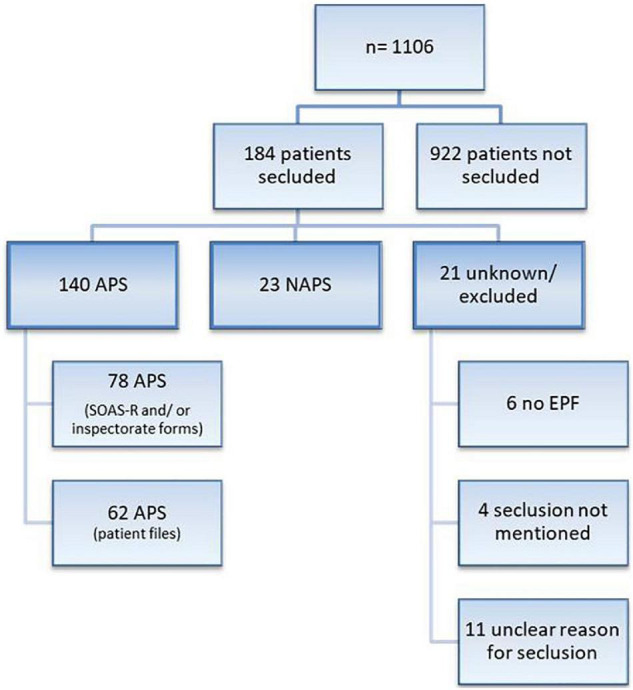
Flowchart of population studied. APS, aggression preceded seclusion; NAPS, no aggression preceded seclusion; SOAS-R, staff observation aggression scale revised; EPF, Electronic Patient Files.

According to the SOAS-R or inspectorate forms, 78 seclusions had been preceded by aggression. On the basis of electronic patient files (EPF), we classified an additional 62 of the remaining 106 cases as APS. We excluded 21 cases (11% of the 184 patients who had been secluded) because neither the SOAS-R forms, inspectorate forms or the EPF contained enough information about the seclusion to classify it as NAPS or APS. There was thus no indication of aggression in 23 of the remaining 163 patients (14%) (Figure 1).

### Aggression Preceding Seclusion vs. No Aggression Preceding Seclusion

Univariable analyses of the factors discriminating between APS and NAPS showed that NAPS was inversely associated with the daytime (7 a.m.–7 p.m., *OR* = 0.38, 95%-CI: 0.15–0.98) and with the first day of hospitalization (*OR* = 0.29, 95%-CI: 0.09–0.89); see Table 1.

**TABLE 1:** Univariable associations between patient, diagnostic, contextual, and aggression characteristics and no aggression preceding seclusion using logistic regression.

	Total^#^		Aggression		No aggression		Test	Statistic		Excluded cases	
	N	%	n	%	n	%	OR	95%CI	p	n	%
Total	184		140		23					21	
**Demographic**											
Female	80	44	61	44	10	44	1.0	0.41–2.4	0.993	9	43
Western, *n* = 179, 97.3%	158	88	120	88	20	91	1.3	0.29–6.2	0.715	18	86
Married, *n* = 146, 79.3%	31	21	23	21	4	20	0.94	0.29–3.1	0.911	4	24
Age^•^ (median IQR)	42.5	30–56	42	29–55.5	51	38–64	*1.0*	*1.0*–*1.0*	*0.096*	41	30–54.5
Age per 10 years							*1.2*	*0.96*–*1.6*	*0.096*		
**Diagnoses**											
Psychotic disorder	95	52	74	53	11	48	0.82	0.34–2.0	0.655	10	48
Substance abuse disorder^°^	12	6.5	10	7.1	0	0.0	–	–	–	2	9.5
Personality disorder	42	23	35	25	3	13	0.45	0.13–1.6	0.219	4	19
**History**											
SOAS-R in year before seclusion	49	27	33	24	6	26	1.1	0.42–3.1	0.793	10	48
Involuntary status in year before seclusion	71	39	55	39	10	44	1.2	0.49–2.9	0.704	6	29
Previous admission(s)	105	57	82	59	13	57	0.92	0.38–2.2	0.853	10	48
**Context of seclusion**											
Open ward (vs. closed ward)	21	11	14	10	4	17	1.9	0.56–6.4	0.301	3	14
Longstay ward (vs. admission ward)	45	25	34	24	5	22	0.87	0.30–2.5	0.791	6	29
Involuntary status ǂ	71	39	53	38	7	30	0.72	0.28–1.9	0.495	11	52
Daytime (7 a.m.–7 p.m.)	93	51	75	54	7	30	**0.38**	**0.15**–**0.98**	**0.045**	11	52
Duration of hospitalization until seclusion in days (median, IQR)	2	0-35.5	1	0–31	3	1–20	1.0	1.0–1.0	0.293	16	1–314.5
Seclusion at first day of hospitalization	66	36	59	42	4	17	**0.29**	**0.09–0.89**	**0.031**	3	14

*^#^Because of missing values the total number of cases could be less than 184. In these cases the exact number of analyzed cases is added.*

*^•^Because this variable is continuous an adjusted OR was calculated for every 10 year increase (age).*

*°Since there were no seclusions without preceding aggression by patients with a substance use disorder an odds ratio could not be calculated.*

*ǂ As involuntary seclusion in the Netherlands needs to be accompanied by an involuntary admission we choose to analyze the juridical status 1 day before the seclusion.*

*OR, Odd’s ratio; CI, confidence interval; IQR, inter quartile range. Bold means p-value < 0.05; Italic means p-value < 0.20.*

Of the remaining variables only age had a *p*-value < 0.20. It was therefore added to the multivariable logistic regression. The multivariable analyses showed that daytime and the first day of hospitalization were both still inversely associated with NAPS. In other words, on the first day and during daytime, more seclusions were related to aggression, while relatively more seclusions for non-aggressive reasons occurred after the first day, and during nighttime (see Table 2).

**TABLE 2:** Multivariable logistic regression model of differentiating characteristics between seclusion with and without preceding aggression, *n* = 163.

	Enter model			Final model		
Characteristic	OR	95% CI	p	OR	95% CI	p
Constant	0.16			0.36		
Age per 10 years	1.2	0.93–1.5	0.168			
Daytime (7 a.m.–7 p.m.)	0.35	0.13–0.93	0.035	0.36	0.14–0.95	0.038
Seclusion at first day of hospitalization	0.30	0.10–0.96	0.042	0.28	0.09–0.86	0.027

### Reasons for Seclusion Without Preceding Aggression

From the 23 NAPS cases, we extracted 50 text fragments specifying reasons for seclusion.

**FIGURE 2:**
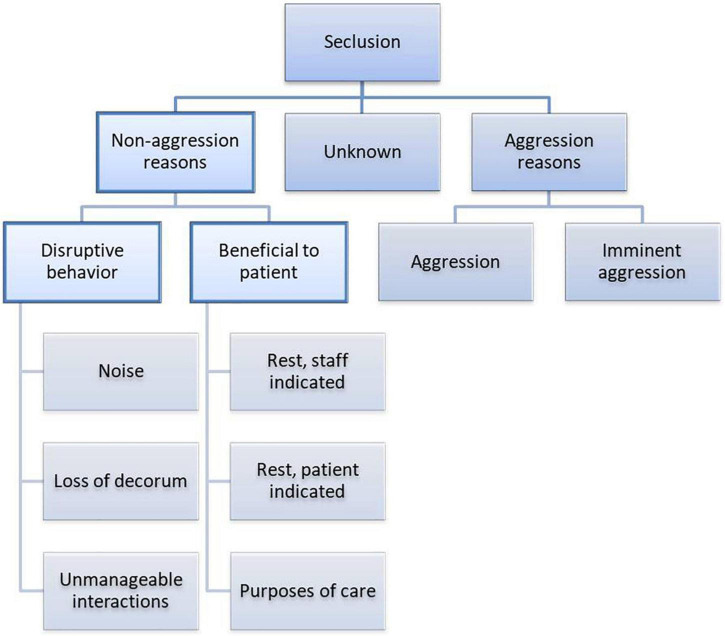
Flowchart of the reasons for seclusion.

The reasons provided for seclusion without preceding aggression fell into two main themes: “disruptive behavior” (29 text fragments, 13 cases) and “expected benefit/beneficial to patient” (18 text fragments, 15 cases). Reasons for seclusion are provided in the flowchart in Figure 2.

#### Disruptive Behavior

The label “disruptive behavior” was used in cases in which a patient’s behavior had disturbed the ward environment, staff members or fellow patients; or when such a disturbance had been imminent. This label—which included agitation—is specified in more detail below.

“Disruptive behavior” included patients’ noisy behavior (such as shouting) especially at night when one awakens other patients with this noise:

*Patient 1: Ms [was] very noisy early in the night. [*…*] She didn*’*t understand she was waking people. By 5 o’clock she [was] screaming again and panicking in the ward. Not a single nurse could get through to her. [As] several clients were awake, [we] decided to place her in t-out [time-out* = *seclusion] after consulting with the chief nurse. [*…*] Once there, she kept on screaming and banging on doors.*

It also included loss of decorum, for example smearing with blood or feces, by walking into other patients’ bedrooms, or by walking around naked:

*Patient 2: Patient came in very animated, very confused and behaving bizarrely. Immediately took off his clothes (uninhibited), wanted to dance, laughed a lot and talked incomprehensibly, rattled on and on. […] He could not be kept in the room, wanted to go into the corridor naked [*…*] Due his extreme restlessness [we] decided after deliberation to seclude him.*

And:

*Patient 3: I saw pt [patient] rubbing the walls in the hallway around 12:45 am. Upon further investigation, it turned out that he was smearing all the walls with a plastic bag with feces. [I] pressed the alarm bell and overpowered him and took him to secl[usion].*

This group also included behavior that became unmanageable when a patient’s interactions with his or her fellow patients and/or staff became bothersome. For example when a patient interfered unwantedly with fellow patients, or was provocative as in the following case:

*Patient 4: Mr. was very tense this afternoon. [He] was very angry with a fellow client who had supposedly stolen his lighter, and he had also pushed her. Received a warning for this. Negative behavior persisted throughout the evening, he showed annoying behavior towards fellow clients, standing nose to nose, making racist remarks, pushing etc. He did not heed further warnings. [*…*] [He] was given a choice between an hour [in his] room or [a] whole night*’*s seclusion. Did not adhere to this rest hour, refused to come along voluntarily, was subsequently compelled to go into seclusion.*

#### Beneficial to Patient

This theme was labeled in cases in which staff or the patient had the impression that the latter *needed* seclusion. In most cases this meant that the patient needed rest. If indicated by staff this was for example because the patient needed to cut out most stimuli, had been behaving restlessly, or was exhausted. This is illustrated by the following text fragments:

*Patient 5: Cl. [client] was proactive, smeared blood around the ward. Advice [of the MD for a] low-stimulus environment.*

And:

*Patient 6: […] A. rested until 3 p.m., but this didn*’*t help. […] She doesn*’*t feel well in her room either, she wants complete rest. When undersigned suggested [the] seclusion [room], she interpreted it negatively, that we wanted to seclude her. In the end she indicated that she wanted to be secluded for 1.5 hours. Which is what happened.*

If patients themselves indicated that they needed rest in the seclusion room, there were various underlying reasons. These were for example the desire to get some sleep, to have a break from restlessness and anxiety, or to feel safe and secure. This is illustrated by the following examples:

*Patient 7: Patient was very friendly this morning. Later on, increasingly suspicious and restless. Wanted to go to the seclusion room at 10.15 to relax.*

And:

*Patient 8: Pt [*…*] was anxious; he said he had been threatened with a knife by 2 or 3 guys, he had then fled into the reception area. I picked him up from reception, [he] did indeed looked scared, wide-eyed, told the story of the guys who were supposed to have threatened him. We walked back to the ward together. On our way we saw 2 boys arriving. According to pt these were the people who had threatened him. Pt tried to run away. When they met us, the young men asked for directions [*…*], they turned out to be calm and nice guys. Pt was suspicious and made some strange comments. Pt remained restless until 2.30 am, somewhat anxious, asked regularly if his family was OK, if his girlfriend was OK, asked for a lot of confirmation. Making agreements on a low-stimulus environment didn*’*t work. Pt turned on the TV loudly. At 2.30 am pt finally decided to go to the seclusion room, indicating clearly that he wanted to go there.*

The last subgroup within this theme involved seclusion for the purposes of care—for instance if seclusion was needed to administer medication, or if the patient needed continuous supervision:

*Patient 9: Ms was brought by ambu[lance] this afternoon by 5.00 pm. At home she had resisted fiercely; paramedics had had a hard time. She had been injected with 4 mg lorazepam and 2.5 mg haloperidol. Very sedated when she arrived, so no interview possible on admission. [We] decided to take her to the secl[usion room] of ward 40. Although she is sedated now, we decided to bring her to the secl[usion room], due to the information of the ambulance personnel. We left the doors open. She is now more in view [of the nurses]. She is also at risk for falling.*

## Discussion

The results of this mixed-methods study showed that approximately 14% of seclusions had not been preceded by aggression or imminent aggression. This type of seclusions was relatively more frequent after the first day of hospitalization and during nighttime. And we grouped the reasons for seclusions without preceding aggression into two main themes: “beneficial for the patient” or “disruptive behavior.” These results are discussed below.

### Number of Seclusions Preceded by Aggression

Relative to the findings in other studies, our finding that 14% seclusions were not preceded by (imminent) aggression is rather low. Though some studies reported even lower rates (22, 25), we found more that reported higher ones (13, 19–21, 23, 24, 26–28). A partial explanation for this is that our use of three sources (rather than one) to identify APS led to a very strict selection of NAPS. While our study relied on multiple sources, including the electronic patient files, to collect information on the reason for seclusion, most other studies used staff questionnaires or specific forms.

It is also possible that the Dutch inpatient population is different from its equivalents in other countries, as the Netherlands has more mental health beds per 100,000 population than most other European countries (45). If, as in other countries, there are fewer beds, admissions may be restricted mainly to patients with unmanageable behavior who are not eligible for treatment at home. If so, this might lead to relatively more aggression in psychiatric hospitals.

Our finding also means that 14% of the seclusions in this hospital took place for reasons other than aggression. As indicated in the introduction, the necessity for seclusion in such cases can be questioned: is the deprivation of a person’s freedom proportionate to the patient’s disruptive behavior or to the possible beneficial effect of seclusion? One might also question whether in these situations seclusion is truly used as a last resort to prevent serious harm. In view of the fact that some patients actually *ask* to be placed in seclusion, our results even suggests that seclusion may be viewed as care as usual.

### Aggression Preceding Seclusion vs. No Aggression Preceding Seclusion

Only two of the factors of the quantitative analyses could discriminate between APS and NAPS: first day of hospitalization and daytime.

Conceivably, this suggests that staff who encounter patient’s aggression at the first day of hospitalization need to act in order to restore patients’ safety and their own. In contrast, if they encounter disturbing behavior, staff may wait to see how it develops, and resort to seclusion later during hospitalization. This may also indicate that some seclusions that are not preceded by aggression take place when nurses with experience of a specific patient decide to seclude that patient before he or she manifests aggressive behavior. However, other characteristics that indicated staff familiarity with the patient in question, such as previous admissions or aggression incidents in the patient’s history, did not differentiate between APS and NAPS.

The other discriminating factor was time of day. At night, relatively more seclusions were not preceded by aggression. This could be explained by disturbing behavior, affecting the sleep and most needed rest of other patients, while fewer staff is available at night. It is easy to understand that nurses separate noisy patients from others at night in order to ensure enough silence for the other patients.

Interestingly and in contrast with Keski-Valkama (23), we found no associations with psychiatric diagnoses.

### Reasons for Seclusion: Qualitative Results

After studying patient files for reasons for seclusion without preceding aggression, we grouped these reasons into two categories: “disruptive behavior” and “beneficial to the patient.”

Reasons for seclusion that were often reported in other studies involved several forms of disruptive behavior, such as agitated, disorganized, escalating, and inappropriate or uncontrolled behavior (19, 20, 22–24, 26, 28, 33, 46). Some of these behaviors might precipitate acts of inpatient aggression (47). In such cases seclusion might have prevented aggressive behavior. On the other hand, in cases of falsely positive labeling disruptive behavior as behavior that precipitates aggression, seclusion is used, while not necessary (48).

There are few studies that found “beneficial to the patient” as a reason for seclusion. Some of these described seclusion at the patient’s request (24, 26, 33, 46), but, unlike in our own study, this was not specified any further, like for example for rest or feeling safe and/or secure.

Although Betemps (20) reported in the context of patient agitation that seclusion was used to reduce the number of stimuli, we found no other studies in which reduced stimuli were claimed to be beneficial. Neither did Betemps’ study contain many instances in which seclusion had been used for this reason.

The literature lacks sound objective evidence for a truly beneficial effect of seclusion. In their review, Chieze et al. (1) stated that “subjective perception has high interindividual variability and can be positive, for example with feelings of safety. However, seclusion and restraint are mostly associated with negative emotions, particularly feelings of punishment and distress.” But conclusions on protective or therapeutic effects of seclusion and restraint were more difficult to draw, and results of their review provide little evidence for these outcomes (1).

### Clinical Implications

As indicated in our introduction, awareness of seclusion patterns, including the reasons for seclusion, can be used to tailor and implement seclusion-reduction interventions (35). Interventions to prevent seclusion could be tailored to the various reasons for seclusion. For example with noise-canceling insulations between patients’ bedrooms, placement in intensive care units away from the patients who are bothered by the behavior, or the use of temporary one-on-one care, the reduction of seclusion for disruptive behavior might be feasible.

Research is needed to explore patients’ motives for requesting seclusion. If, for example, patients wish to decrease stimuli, there are options for doing so in their own bedroom, or for creating a room that soothes the senses, such as a comfort room (49, 50), or for placement in an empty room, that the patient can always leave whenever they wish. At the same time, it should also be established whether reducing stimuli is indeed beneficial: there are indications that sensory deprivation leads to psychotic-like symptoms in healthy people (51).

If we assume that our finding of a low percentage of seclusions for non-aggressive reasons is true for all psychiatric hospitals, the greatest reduction in the use of seclusion may be achieved by reducing aggression itself. That could start with identifying potential aggression at an early stage, as Jayaram et al. (52) did with the Phipps aggression screening tool (52), or Abderhalden et al. (53), Van der Sande et al. (54), and Blair et al. (55) with the Brøset Violence Checklist. However, not all aggression-screening studies have been effective (56), and a recent study suggested that aggression in mental health hospitals may be more situation-specific and less a factor of mental illness (57). Due to the circumstances of COVID-19, Martin et al. (57) focused on proactive co-design (i.e., the influence of staff and the representatives of family and patients), which led unexpectedly to less aggression and less use of coercive measures on the wards (57). As stated in the field norms formulated by professionals and patients (58), various contextual factors are important to reduce the use of coercive measures. They include staffing levels that allow enough nurses per bed, options for increasing care to one-on-one guidance, enough space per patient, and enough activities during the day, also in the weekend. With others, these factors have been incorporated into a model fidelity scale developed for High Intensive Care units in psychiatric clinics, the HIC monitor (59). Van Melle et al. (60) showed that high fidelity to the HIC monitor led to lesser use of coercive measures (60). If these factors are not well addressed, staff may easily resume the use of coercive measures. The intervention “first 5 min of the admission process” (also incorporated into the HIC monitor) focuses specifically on preventing aggression and seclusion during the first hours of hospitalization (61). Another focus to reduce the use of seclusion was suggested by Doedens et al. (62): Because nurses currently view coercive measures as “undesirable, but necessary” for dealing with aggression, mental health care could protect patients from the unnecessary use of coercive interventions by improving perceived safety by nurses and their familiarity with alternative interventions.

### Strengths and Limitations

The three main strengths of this study are (1) its use of three sources for detecting any aggression, which ensured that NAPS is truly free of aggression; (2) its combination of quantitative and qualitative methods, which provided several points of view on this topic; and (3) its use of electronic patient files, which ensured that the data are in conformity with normal daily clinical care.

This approach, based on daily practice, also created a potential limitation: the possibility that the files and forms from which we collected information were incomplete, as they had not been filled out for the purposes of our research, but as part of the primary process of caring for patients, for the hospital’s safety monitoring, and to account to the inspectorate for any uses of coercion.

Even though they provided a considerable amount of information, our use of these three sources, each with its own purpose, also provided different, and even potentially contradictory, views of the incidents or seclusions in question. To account for cases of seclusion, reports to the inspectorate are prone to a certain exaggeration, whereas incidents of aggression may be underreported, as nurses may not have witnessed every incident. This may be compounded by the scope for subjective interpretations in definitions of aggression, mainly in descriptions of *imminent* behavior that was threatening to self, others or property. For example, even if there is no threat or actual aggression, members of certain groups may perceive members of other groups as threats simply due to their group membership and the ways we are socialized to fear the “other” (63).

The tumult of the day, especially with seclusion and/or aggression incidents, may easily lead to underreporting in nurses’ daily reports. And second-hand reporting may result from nursing staff having too little time to write a thorough report and therefore ask staff on the next shift to report for them. In cases of seclusion without preceding aggression, it is also possible that some nurses are hesitant to record the reason for seclusion.

As we were unable to relate aggression incidents reported by the SOAS-R to the actual time of seclusion, we assumed that the reason for a particular case of seclusion was any aggression reported by the SOAS-R on the day seclusion took place. This may mean that the aggression had also taken place in response to the initiation of the seclusion, or during the period in seclusion. In other words, it is possible that seclusion had caused the aggression rather than vice-versa. We nonetheless believe that most of these cases involved signs—overt or otherwise—of the imminent aggression that had caused the initiation of the seclusion in the first place.

Another limitation of our data is the number of excluded cases of 11%—a number almost as large as the number of NAPS. Data on these might have changed the ratio of NAPS to APS, though the excluded cases have a profile that is neither typical for APS nor for NAPS. We have 3 potential explanations for the lack of information on these seclusions: First, as the last column in Table 1 illustrates, this group stands out from the included cases in that they were more often admitted to long stay wards, had longer admission duration, and were more often admitted involuntarily. These factors may indicate that these cases concern patients who are long term residents of the clinic, with well-known behavioral patterns. This may result in underreport of daily notes, including incidents like aggression and coercive measures. It might be possible that this underreporting occurred more often in NAPS than APS. Second, just before the start of this study, the electronic patient files were implemented (instead of the paper patient files). Though most of our cases were documented in the EPF, six cases were not yet. Unfortunately we were unable to find the daily notes of the paper files of these cases. It is unlikely that this relates to either APS or NAPS. Third, in four cases seclusion was not mentioned in the EPF on the day mentioned on the seclusion form. We hypothesize that in these cases the date of the seclusion is probably noted wrongly on the form. Hence, it is not possible to match it with data from the EPF on the seclusion. This too, is unlikely to relate to either APS or NAPS.

We found some striking differences between de excluded cases and the total group. For example, we observed a higher percentage number of previous aggression incidents in the year before the seclusion (48 vs. 27%), but this might be a bias. We already concluded that these patients were admitted longer than the non-missing cases. Consequently, they were also longer “at risk” to be exposed to some form of aggression. Aggression incidents of patients in the year before the seclusion while not being admitted, are not registered nor counted. We are unable to verify any of this kind of incidents outside the hospital.

In this study we analyzed EPF text fragments from all available NAPS cases in one hospital setting. Within this approach, contrasting with other qualitative approaches, we did neither strive for, nor reach saturation. Further studies on NAPS in other settings can potentially elaborate our framework for reasons for seclusion. And as we did not systematically check all reasons for seclusion, but only those for NAPS, we cannot interpret the number of cases stated in the qualitative part with studied reasons for seclusion as a quantitative measure.

The hospital in question started its seclusion-reduction program in 2006. This program focused on improving hospitality including the use of a comfort room (49, 50), which can be used for sensory soothing. Although we did not study this, it might have led to a reduction of NAPS more than APS.

Another limitation is that our data are about 10 years old. After checking, however, we established that the numbers of seclusion and aggression incidents in this hospital in 2008 and 2009 were comparable to those in 2018 and 2019. At a national level, seclusion in 2019 was still an important measure that was still being used more often than other coercive measures. And the total number of seclusions at a national level was in this year largely the same as at the end of the seclusion-reduction programs in 2012 (64). Our findings are thus likely to retain their clinical validity.

We performed a considerable number of statistical analyses, only few of which were statistically significant. It is possible that these findings may have been the result of a type 1 error (i.e., rejecting the null hypothesis when it’s actually true).

Finally, the generalizability of our results is limited by the fact that our study was conducted in a single hospital in the Netherlands. Before the study started, this hospital had almost completely banned the use of mechanical restraints, which were still being used occasionally, but only on the geriatric wards. We can therefore assume that if coercion is used in this hospital, it is almost always seclusion.

## Conclusion

Interventions on reducing the use of seclusion may benefit from an awareness of the different reasons for seclusion. As our thorough examination of various sources showed that little seclusions had not been preceded by aggression, interventions intended to prevent aggression, or to handle aggression by other means than by seclusion, should have a considerable effect on reducing the use of seclusion. However, attention should also be paid to the remaining reasons for seclusion, such as handling disruptive behavior and focusing on the beneficial effects of reduced stimuli or continuous guidance without locking patients up alone in an empty room. Future research on interventions to reduce the use of seclusion should therefore not only aim to reduce seclusion, they should also analyze whether seclusion for certain reasons is reduced more than seclusion for other reasons.

Our findings indicate that the reasons for secluding psychiatric inpatients are complex and varied. As each type of seclusion, whether preceded by aggression or not, requires a different management approach, it may be important to characterize the reasons for seclusion when determining which interventions should be implemented to reduce its use.

## Data Availability Statement

The raw data supporting the conclusions of this article will be made available by the authors, without undue reservation.

## Ethics Statement

Ethical review and approval was not required for the study on human participants in accordance with the local legislation and institutional requirements. Written informed consent for participation was not required for this study in accordance with the national legislation and the institutional requirements.

## Author Contributions

FV, JV, EN, HN, and CM conceived and designed the study. FV collected the data. FV and EN rated the first data into APS and NAPS. FV analyzed the quantitative part of this research that was closely advised by EN. FV and JV performed the qualitative part of this study. FV wrote the first and subsequent drafts, which were revised critically for important intellectual content by JV, EN, and CM. All authors approved the final version.

## Conflict of Interest

The authors declare that the research was conducted in the absence of any commercial or financial relationships that could be construed as a potential conflict of interest.

## Publisher’s Note

All claims expressed in this article are solely those of the authors and do not necessarily represent those of their affiliated organizations, or those of the publisher, the editors and the reviewers. Any product that may be evaluated in this article, or claim that may be made by its manufacturer, is not guaranteed or endorsed by the publisher.

## Abbreviations

APS: aggression preceding seclusion
NAPS: no aggression preceding seclusion
SOAS-R: Staff Observation Aggression Scale Revised
EPF: Electronic Patient Files.
